# Case report: Emphysematous cystitis due to *Escherichia coli* infection with the extension of gas into multiple locations in two non-diabetic dogs: a computed tomographic diagnosis and successful management

**DOI:** 10.3389/fvets.2023.1196006

**Published:** 2023-07-14

**Authors:** Eun-Ji Lee, Jeong-Min Lee, Jin-Young Kim, Tae-Sung Hwang, Kun-Ho Song, Joong-Hyun Song

**Affiliations:** ^1^Department of Veterinary Internal Medicine, College of Veterinary Medicine, Chungnam National University, Daejeon, Republic of Korea; ^2^Institute of Animal Medicine, College of Veterinary Medicine, Gyeongsang National University, Jinju, Republic of Korea

**Keywords:** dog, *Escherichia coli*, emphysematous cystitis, ischiorectal fossa, urinary tract infection

## Abstract

Emphysematous cystitis is an extremely rare, complicated urinary tract infection with the presence of gas in the bladder wall and lumen caused by gas-producing bacterial infections. A 7-year-old spayed female pomeranian dog was presented with a 3-day history of hematuria and pollakiuria (case 1), and a 9-year-old spayed female jindo dog was presented with a 4-day history of intermittent hematuria (case 2). Imaging modalities, including radiography, ultrasonography, and computed tomography, and bacterial culture tests were used for the diagnosis. Emphysematous cystitis due to *Escherichia coli* infection with the extension of gas into multiple locations was identified in both cases. Based on the results of antibiotic susceptibility testing, systemic antibiotics were initiated. Both animals had an excellent response to antibiotic treatment, and the clinical signs of the gas collection were completely resolved within ~1 month after treatment initiation. This response was sustained without recurrence in the follow-up period. This case report describes clinical details of extremely rare canine cases of emphysematous cystitis with the extension of gas into multiple locations and evaluates the clinical efficacy of antibiotic therapy.

## Introduction

Urinary tract infection (UTI) is a common condition affecting ~14% of dogs at least once in their lifetime (1). UTIs are either classified as complicated—occurring in conjunction with functional or anatomical abnormalities, or uncomplicated—presenting sporadically in otherwise healthy animals (2). The diagnosis of UTI is based on clinical signs, diagnostic imaging, and bacterial urine culture results (3). Antibiotic therapy based on urine culture and antibiotic susceptibility is the mainstay of treatment (3). Emphysematous cystitis (EC) is a rare, complicated UTI caused by gas-forming bacteria (4). EC is uniquely identified based on the presence of gas in the bladder wall and lumen (4). In humans, it occurs mostly, but not exclusively, in patients with diabetes mellitus (DM) (5). Although some predisposing factors are known, including chronic UTIs, indwelling urethral catheters, neurogenic bladder, urinary tract outlet obstruction, and immune deficiency, the pathogenesis of EC in patients without DM is not yet fully understood (5, 6). Urinary glucose and tissue proteins are used as fermentation substrates by gas-forming bacteria (6). *Escherichia coli* is the most commonly isolated organism in EC (7, 8). Clinical signs are non-specific, including hematuria, dysuria, stranguria, pollakiuria, abdominal pain, and urinary incontinence (8). Imaging methods, such as abdominal radiography, ultrasonography, and computed tomography (CT), are usually required for a conclusive diagnosis (8). CT is a widely accepted and well-known diagnostic tool for EC in humans but has not been used as commonly utilized in veterinary medicine.

If EC progresses to involve the kidney, emphysematous pyelonephritis (EPN) occurs. It is characterized by the accumulation of gas in the kidney parenchyma, collecting system, or perinephric tissues (9). EPN shares many characteristics with EC in terms of pathogenesis, clinical presentation, diagnosis, and treatment. Delayed intervention in EC leads to EPN (9, 10). In the limited reports of EPN in veterinary patients, affected animals had unfavorable outcomes, suggesting that EPN has a poorer prognosis than EC in this population (11).

In this report, we spotlight EC within veterinary medicine, specifically in non-diabetic patients. A primary focus is the successful utilization of antibiotic therapy in managing EC, emphasizing the importance of early intervention and accurate diagnosis. We delve into the complexities of gas presence in non-typical locations beyond the urinary bladder, underscoring the potential for EC to manifest diversely. Furthermore, we highlight the critical role of CT in diagnosing EC, in contrast to the limitations of ultrasonography in identifying these atypical presentations. To this end, we present two cases of EC in non-diabetic dogs, including one instance of the seldom reported subcutaneous emphysema, showcasing the varied presentations and challenges in diagnosing EC. This evaluation aimed to enrich the understanding and management of EC in veterinary medicine.

## Case report

### Case 1

A 7-year-old spayed female Pomeranian with a history of recurrent UTI presented with hematuria and pollakiuria for 3 days. The patient had undergone surgical correction of left medial patellar luxation and left radius corrective osteotomy 6 and 8 years ago, respectively, and recently showed skipping gait of the right hindlimb. In addition, 45 days before presenting, she underwent bilateral regional mastectomy and ovariohysterectomy. During hospitalization, a urinary indwelling catheter was used to support urinary elimination. After discharge, the owner refrained from taking the dog for walks for ~1 month, despite the dog being habituated to urinating outdoors. Furthermore, owing to the dog's discomfort with movement, the frequency of urination at home during this period was less than twice per day.

**Figure 1:**
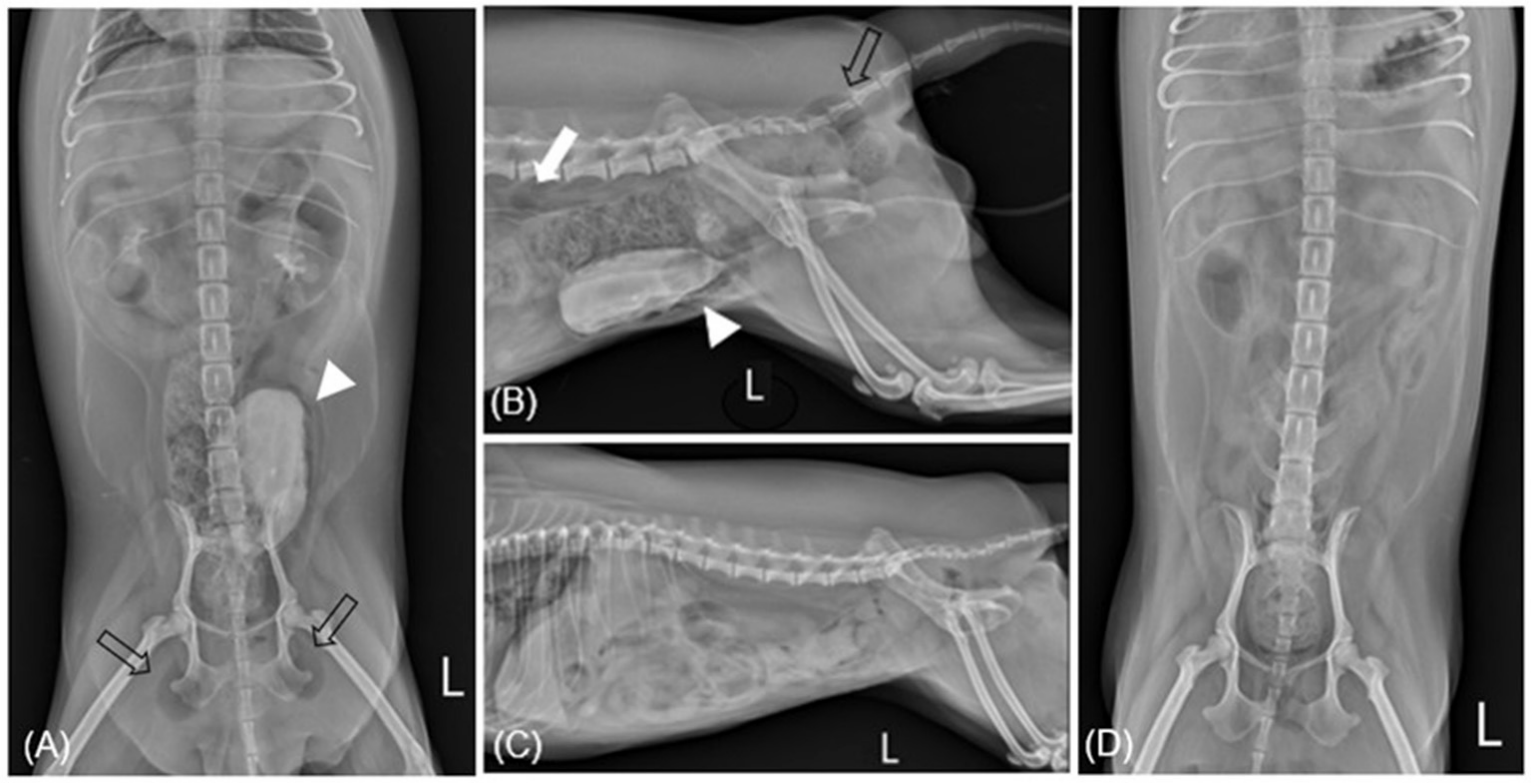
Radiographic imaging before **(A, B)** and after **(C, D)** treatment of case 1. **(A)** After retrograde urography (RGU), ventrodorsal radiography reveals gas within the bladder wall (allow head) and subcutaneous emphysema: ischiorectal fossa (empty arrow). **(B)** After RGU, there are multiple gases consistent with pneumoperitoneum: retroperitoneum (white arrow). **(C, D)** Show overall resolution after undergoing antibiotic treatment for 4 weeks. L, left.

**Figure 2:**
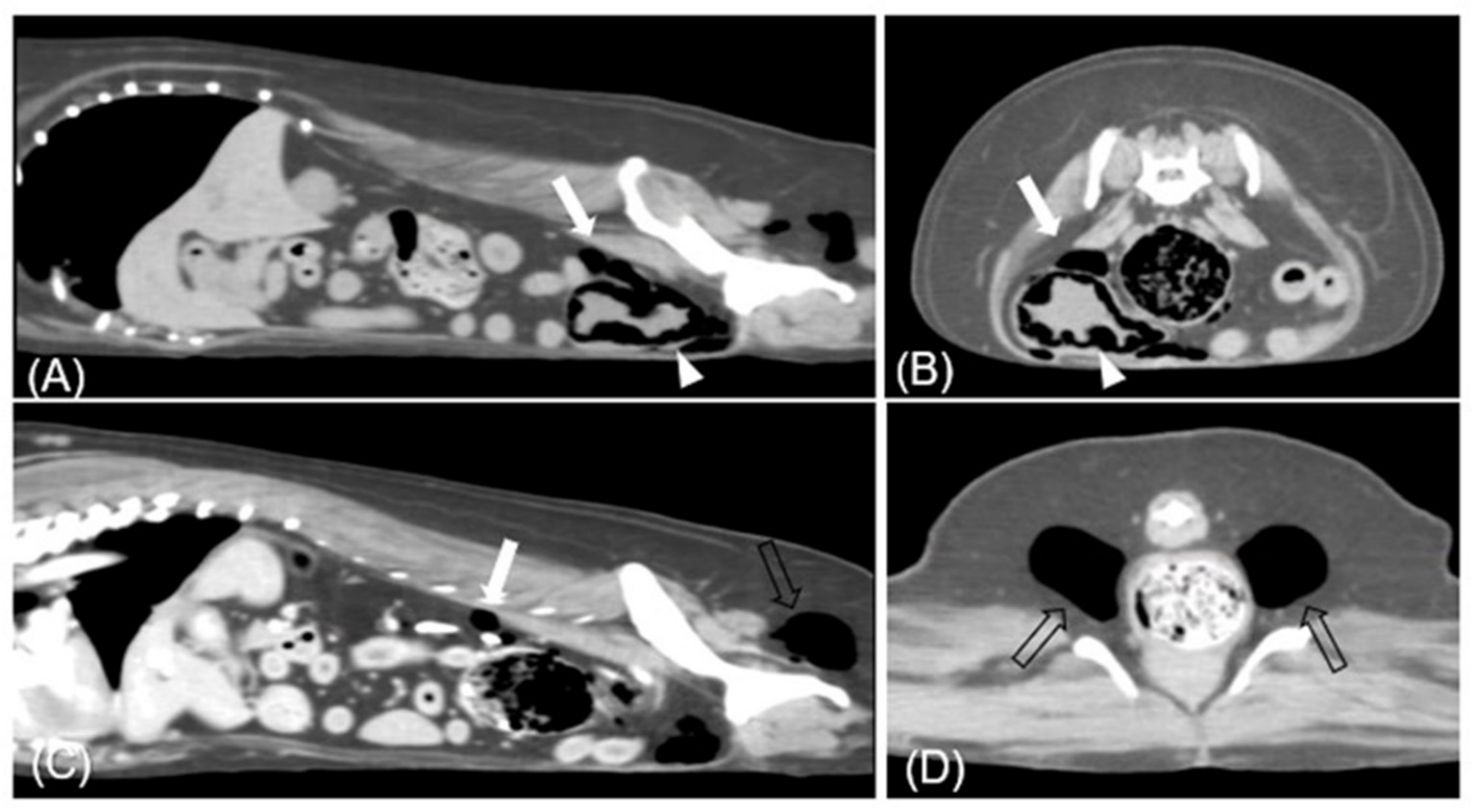
Transverse **(A, C)** and sagittal **(B, D)** CT images (soft tissue window) of case 1. Each presents gases in **(A, B)** urinary bladder base level, **(C)** retroperitoneum level, and **(D)** ischiorectal fossa. Multiple gases are found around the bladder wall and within the bladder (arrowhead). The largest bubble within the bladder wall is 3 mm. Various sizes of gas bubbles are presented in the retroperitoneum (white arrow). In bilateral ischiorectal fossa, each has ~1.5 cm gas (empty arrow).

On presentation, she was alert and generally well-appearing. Physical examination revealed normal rectal temperature, pulse, respiratory rate, effort, and blood pressure. There was no evidence of toxic exposure or infection. Blood analysis, including complete blood count (CBC; ProCyte Dx^®^, IDEXX, ME, USA), electrolytes (pHOx Ultra; Nova Biomedical, MA, USA), and serum biochemistry (Catalyst One, IDEXX, ME, USA), was unremarkable with no evidence of DM. Free gas in the pneumoretroperitoneum and bilateral gluteal area was suspected on abdominal radiography (MDXP-40, MEDIEN, Korea) (Figures 1A, C). Ultrasonographic (iU22^®^, Philips, USA) examination of the urinary system was obscured by gas-induced artifacts; hence, CT scanning for more precise evaluation was recommended. CT (Alexion, Toshiba Medical Systems, Japan) scanning revealed multiple pockets of gas in the bladder wall, pneumoperitoneum, around the bladder, the posterior abdominal cavity, and the subcutaneous tissues around the ischiorectal fossa (Figure 2). Urinalysis obtained by catheterization revealed a straw and clear urine appearance, a specific gravity of 1.037, pH 9, trace protein, and 2+ blood. Microscopic examination revealed some red blood cells (RBCs), white blood cells (WBCs), and epithelial cells. Urine sedimentation with Diff-Quick staining revealed numerous rod-shaped bacteria, RBC, and neutrophils. A urine culture identified *Escherichia coli* as susceptible to both amoxicillin/clavulanic acid (AMC) and enrofloxacin. Based on the imaging findings and the presence of *Escherichia coli* in the urine, the patient was diagnosed with EC with pneumoperitoneum (retroperitoneum and lower abdominal cavity) and subcutaneous emphysema (ischiorectal fossa).

The patient was hospitalized for 3 days to receive parenteral drugs and to undergo close monitoring owing to the risk of bladder perforation resulting from a fragile bladder wall. Antibiotic treatment, consisting of AMC (Amoclan, Hanmi Pharm Co., Ltd., Seoul, Korea; 12.5 mg/kg intravenously every 12 h) and enrofloxacin (Baytril^®^, Bayer Korea; 5 mg/kg intramuscularly every 24 h), was provided during this period. During the period of intensive observation, while the patient was hospitalized, no evidence of bladder perforation was found.

We conducted daily imaging to assess gas dissipation. On the second day, there were no significant clinical changes; however, bladder wall gas partially resolved on radiography. By the third day, bladder wall gas had almost completely resolved, and residual gas around the bladder, in the bladder lumen, and in subcutaneous regions was gradually decreasing. Repeat urinalysis at this point showed no bacteria. The patient was discharged, and oral treatment with enrofloxacin (10 mg/kg orally every 24 h) and AMC (12.5 mg/kg orally every 12 h) was then administered for 4 weeks. The patient returned to the hospital after 1, 2, and 4 weeks for abdominal radiography, ultrasound, and complete urinalysis. Though most gases resolved relatively quickly, the subcutaneous gas in the ischiorectal fossa took at least 3 weeks to resolve. After complete EC resolution, the patient discontinued antibiotic treatment (Figures 1B, D). The patient showed sustained improvement without recurrence in the following 10 months.

### Case 2

A 9-year-old spayed female Jindo dog with a 2-year history of recurrent UTIs and intermittent urinary incontinence presented with 4 days of intermittent hematuria. No other urological symptoms, such as stranguria or pollakiuria, were observed. In the last 2 years, the patient had undergone three surgeries to remove Patnaik grade II mast cell tumors from her left hindlimb. Moreover, after an ultrasound revealed a bladder mass, a partial cystectomy had been performed. Histological examination of the bladder tissue revealed polypoid hyperplasia and mucosal inflammation. *Escherichia coli* had been identified in a urine culture performed 7 months prior to her presentation to our service. Although AMC (12.5 mg/kg orally every 12 h) was administered for *Escherichia coli* cystitis at that time, there was no significant improvement in the condition.

**Figure 3:**
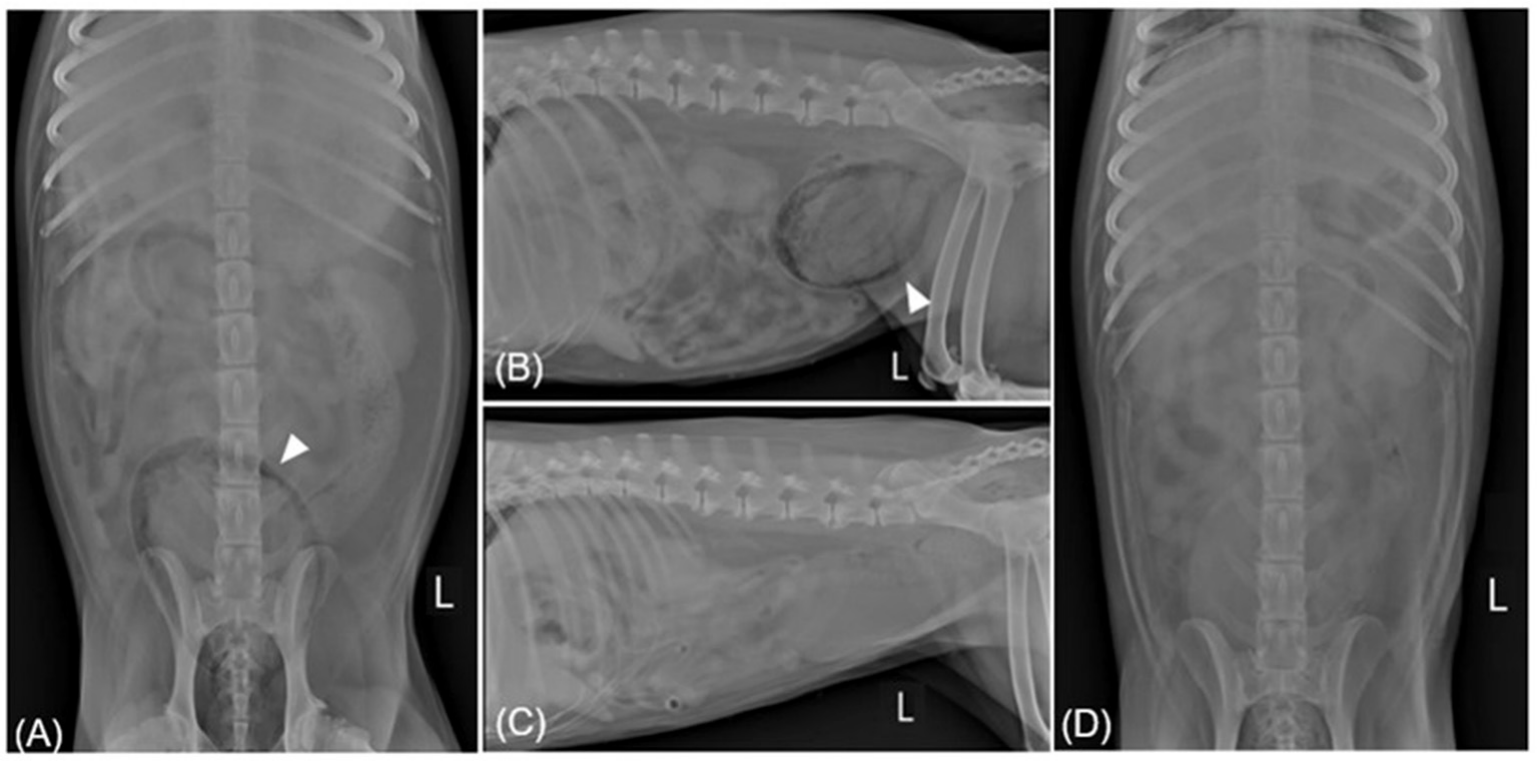
Radiographic imaging before **(A, B)** and after **(C, D)** treatment of case 2. **(A, B)** Ventrodorsal and left lateral radiography reveal gas within the bladder wall (allow head). In spite of the patient being diagnosed with EPN through CT, radiography shows both kidneys to be normal. **(C, D)** Showing overall resolution after undergoing antibiotic treatment for 18 days. L, left.

**Figure 4:**
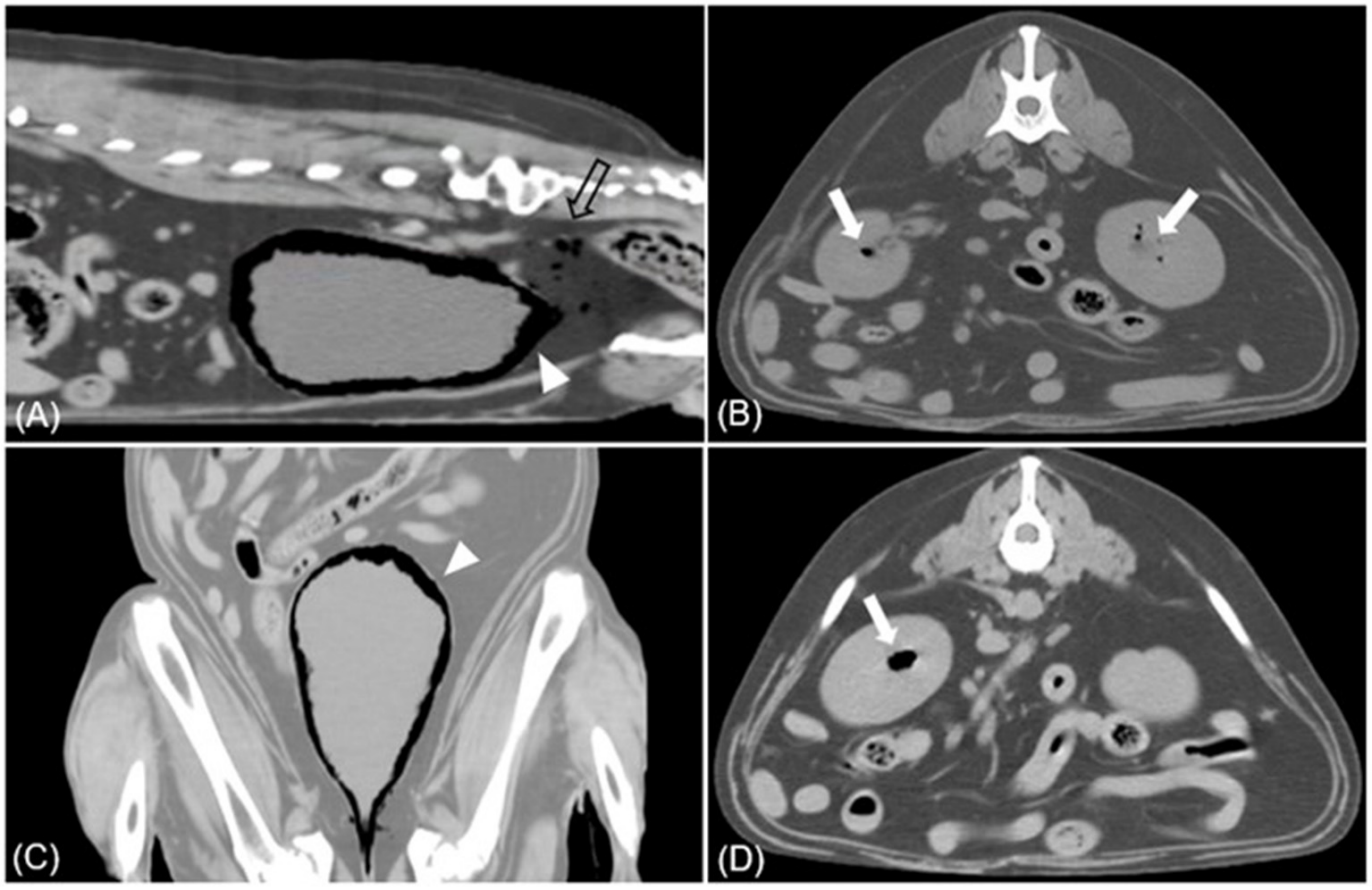
Sagittal **(A)**, dorsal **(C)**, transverse **(B, D)** CT images (soft tissue window) of case 2. **(A, C)** Multiple gases are found around the bladder wall and within the bladder (arrowhead). Various sizes of gas bubbles are present in the pelvic cavity posterior to the bladder (empty arrow). **(B, D)** The accumulation of gases within the parenchyma of both kidneys is found, presenting EPN (white arrow).

Physical examination revealed a normal rectal temperature, pulse, respiratory rate, and blood pressure. CBC revealed mild anemia [hematocrit 30.2%; reference interval (RI), 37.0–61.0%], significant leukocytosis (41.10 × 10^3^/μL; RI, 5.05–16.76 × 10^3^/μl), and significant thrombocytosis (1,014 × 10^3^/μl; RI, 148–484 × 10^3^/μl). There was a predominant neutrophilia (37.81 × 10^3^/μl; RI, 2.9–11.6) and monocytosis (3.185 × 10^3^/μl; RI, 0.16–1.12 × 10^3^/μl), with a relative lymphopenia (0.1 × 110^3^/μl; RI, 1–5 × 10^3^/μl). The serum biochemical profile showed elevated alkaline phosphatase (1,935 U/L; RI, 23–212 U/L), alanine aminotransferase (535 U/L; RI, 10–125 U/L), aspartate aminotransferase (234 U/L; RI, 0–50 U/L), and gamma-glutamyl transferase (195 U/L; RI, 0–11 U/L). Abdominal radiography revealed a moderately expanded bladder and a gas opacity along the bladder wall (Figures 3A, C). The gas opacity and soft tissue density within the bladder were heterogeneous. Reverberation artifacts made it difficult to clearly evaluate the bladder lumen and surrounding structures on ultrasound. A comet tail artifact was observed in the right kidney. The caudal pole of the left kidney was irregular, and there were focal areas of low-echoic parenchyma. The size of both kidneys was within the normal range. On CT, a large amount of gas was detected around the bladder and some areas of the bladder wall (Figure 4), causing a loss of continuity in the bladder wall. There was little possibility of bladder rupture. Additionally, several gas bubbles were identified in the posterior pelvic cavity of the bladder and the abdominal cavity on the right side of the bladder. Gas bubbles were found in both renal pelvises. Complete urinalysis obtained by cystocentesis under ultrasound guidance revealed a yellow and clear urine appearance, a specific gravity of 1.015, pH 5, trace protein trace, and 4+ blood. Microscopic urine examination revealed the presence of RBC, WBC, and rod-shaped bacteria. Urine sedimentation with Diff-Quick staining revealed numerous rod-shaped bacteria, RBC, and neutrophils. A urine culture identified *Escherichia coli* as sensitive only to imipenem, polymyxin B, and amikacin. Based on the bacteriuria and imaging findings, the patient was diagnosed with EC with pneumoperitoneum (lower abdominal cavity and pelvis cavity) and bilateral EPN.

Prior to antibiotic susceptibility results, the patient was administered AMC (12.5 mg/kg orally every 12 h) and enrofloxacin (10 mg/kg orally every 12 h) as empirical therapy. A week later, a microscopic examination of a urine sample obtained via a catheter still showed numerous rod-shaped bacteria and neutrophils. Abdominal radiography revealed no noticeable gas-related changes. Recognizing the insufficient clinical response, the decision was made to hospitalize the patient and implement a therapeutic change, replacing the previous medication with meropenem (Meropenem, Dongkwang, Korea; 9.5 mg/kg intravenously every 8 h) during the 8-day hospitalization period. Upon discharge after 8 days, gas had resolved from all locations, except within the urinary bladder where only a small amount of retained gas was shown on abdominal radiography. Three days later, abdominal radiography showed complete gas resolution (Figures 3B, D), and abdominal ultrasound confirmed remission. Antibiotic treatment was discontinued after 18 days, and phenylpropanolamine hydrochloride (PPA; Propalin^®^, Vetoquinol, UK; 1 mg/kg orally every 12 h) was prescribed to control urinary incontinence. In the following 3 months, the dog showed clinical improvement without the recurrence of EC or EPN.

## Discussion

EC is characterized by the presence of gas in the bladder wall and lumen as a result of gas-producing bacterial infections, especially *Escherichia coli* (12). *Escherichia coli*, implicated in both of our cases, colonizes the gut and is shed in feces, which leads to the colonization of the periurethral area. From here, it can ascend through the urethral tract into the bladder, resulting in continuous asymptomatic reseeding of the urinary tract (13). DM is known as a major risk factor for EC in humans, present in almost 70% of patients (14). High levels of glucose play a key role in EC pathogenesis because glucose is an important substrate for fermentation by gas-forming bacteria (14). Typically, gas produced by bacteria from mixed acid fermentation of tissue glucose is removed by the bloodstream (15). However, local inflammation increases intracellular pressure, decreases tissue perfusion, and allows for the formation of gas chambers as the gas expands in the infected tissue (15). As gases are produced and collect in the bladder, they pass into the submucosa, are released into the infra-peritoneal space around the base of the bladder and may diffuse through and across the abdominal musculature (12). During this process, they can descend into the ischiorectal fossa, as in case 1, or ascend into the kidney, as in case 2 (12).

A retrospective study of 36 dogs with EC found a higher occurrence in middle-aged and older dogs, with only 10.5% of the cases linked to DM. This differs from human cases, where a 2:1 female preponderance and over 50% DM linkage are observed. Despite a higher incidence of UTIs in female dogs, there is no gender bias for EC in animals; it occurs equally in males and females (6, 8). In our report, both dogs were middle-aged, spayed females, and non-diabetic. Although the pathogenesis of EC without DM is not fully understood, glucose in urine and tissue proteins are used as fermentation substrates (6). Along with DM, there are several other risk factors, such as chronic UTI, urinary tract obstructions, morphologic abnormalities, neurogenic bladder dysfunction, indwelling urethral catheters, and immunosuppression (16). In case 1, the patient had an indwelling urinary catheter placed several weeks before the EC diagnosis. In addition, the patient was not walking outdoors for ~1 month and had reduced mobility indoors owing to having undergone several surgeries on the left hindlimb and having developed a skipping gait of the right hindlimb. All these factors combined to reduce urination frequency, which in turn may have led to urinary retention and the failure to effectively eliminate bacteria from the urinary tract (17). Based on this case, we propose that any factor leading to urinary retention can contribute to the development of EC. In case 2, the dog had been suffering from a chronic UTI and 2 years of intermittent urinary incontinence. Moreover, the dog had polypoid hyperplasia and inflammation of the bladder mucosa, resulting in a partial cystectomy. Conditions such as these that affect the urinary system are also thought to be risk factors for EC.

Human mortality from EC reportedly is between 7 and 9.4%, with delayed diagnosis leading to EPN, sepsis, and bladder rupture implicated in 20% of mortal cases (18). Bladder perforation has been documented in five human patients (4.42%, 5/113) (4). Although EC in animals has not been studied as extensively as in humans, bladder perforation may play a similar role; hence, early EC detection is imperative. Blood tests, including CBC and serum biochemistry, are often non-specific in the workup of EC; thus, urinalysis and urine culture are necessary to identify pathogens and choose appropriate antibiotics (1–3, 19). CT is the most reliable tool to differentiate between EC and other conditions, such as colovesical fistulas, with 100% sensitivity and specificity (4, 16, 20). Typical EC findings on CT include a radiolucent line of gas surrounding the bladder appearing like cobblestones or beads, irregular thickening of the mucosa, and free gas within the bladder (16). In both of our cases, radiography was only capable of identifying large amounts of bladder gas, whereas CT enabled the detection of smaller amounts of gas, including those in areas that were beyond the limits of radiography, such as the lower abdomen and retroperitoneum. Additionally, in case 2, CT confirmed EPN whereas radiography showed a normal kidney appearance. Therefore, when there is a possibility of EC, CT is preferred to abdominal radiography. Moreover, if radiography reveals bladder gas, CT scanning should be promptly performed to detect the exact extension of gas.

When humans are hospitalized with EC, systemic antibiotics are usually administered intravenously for 10 days, with an average hospital stay of 7 days. Outpatient therapy typically involves oral antibiotics for 2–3 weeks (6). However, there is no consensus regarding the optimal duration of antibiotic treatment (6). In veterinary medicine, the duration of medical treatment is similarly unclear but generally lasts for 4 weeks (8, 16, 21). We opted for a 4-week antibiotic regimen for EC, as per previous studies, and the prescription of a 28-day systemic antibiotic course in case 1 resulted in complete gas resolution. In addition to the use of antimicrobial agents, we also attempted to mitigate against other EC risk factors. In an effort to relieve urinary retention, we encouraged frequent dog walking to achieve urine emptying at least three times daily, both pre- and post-admission. In case 2, the dog was chronically infected with a multi–drug-resistant *Escherichia coli* strain. Therefore, we had to switch antibiotics during treatment, and a total 18-day course of systemic antibiotics was administered. We decided to discontinue the therapy once radiography confirmed complete gas remission and prescribed PPA to reduce urinary incontinence. In both cases, antibiotics were selected based on susceptibility testing, and the duration of treatment was between 3 and 4 weeks, as in other reports. Although each dog's antibiotic treatment will vary in duration depending on their individual conditions, it will generally be longer than that in an uncomplicated UTI. Therefore, antibiotics should be selected based on the implicated pathogen's antibiotic susceptibility, and serial radiographs should be taken to monitor the treatment outcome. It is also important to concurrently manage any disorders affecting the urinary tract. Prospective studies with larger groups of dogs would help to further refine treatment recommendations.

## Conclusion

EC is a rare form of complicated UTI caused by gas-forming bacteria. Unlike in humans, EC in dogs more commonly affects non-diabetics; thus, clinicians should maintain a high index of suspicion in patients with UTI signs, especially when a gas-forming bacterium is identified. EC can progress to involve a wide range of organs, as in our report of subcutaneous emphysema and EPN. Early diagnosis, preferably through CT, is crucial to prevent such complications. Prolonged administration of antibiotics, based on pathogen susceptibility, along with the management of underlying urinary tract disorders and potential contributing factors, is vital for preventing EC recurrence.

## Data availability statement

The original contributions presented in the study are included in the article/supplementary material, further inquiries can be directed to the corresponding author.

## Ethics statement

Ethical review and approval was not required for the animal study because the owner of the dog signed a written consent. Written informed consent was obtained from the owners for the participation of their animals in this study.

## Author contributions

E-JL wrote the manuscript and contributed to the clinical evaluation, diagnosis, treatment, and follow-up of the patient. J-ML, J-YK, and T-SH contributed to the manuscript design and supervised the manuscript. J-ML wrote the original draft of the manuscript. J-HS supervised the clinical evaluation, diagnosis, and treatment of dogs and revised the manuscript. All authors read and approved the final manuscript.

## References

[1] Ling GV. Therapeutic strategies involving antimicrobial treatment of the canine urinary tract. J Am Vet Med Assoc. (1984) 185:1162–4. 6392247

[2] Teh H. A review of the current concepts in canine urinary tract infections. Aust Vet J. (2022) 100:56–62. 10.1111/avj.13127 34775603

[3] Wong C Epstein SE Westropp JL. Antimicrobial susceptibility patterns in urinary tract infections in dogs (2010-2013). J Vet Intern Med. (2015) 29:1045–52. 10.1111/jvim.13571 26133165 PMC4895361

[4] Ranjan SK Navriya SC Kumar S Mittal A Bhirud DP. Emphysematous cystitis: a case report and literature review of 113 cases. Urol Ann. (2021) 13:312–5. 10.4103/UA.UA_61_20 34421272 PMC8343293

[5] Lobetti RG Goldin JP. Emphysematous cystitis and bladder trigone diverticulum in a dog. J Small Anim Pract. (1998) 39:144–7. 10.1111/j.1748-5827.1998.tb03620.x 9551384

[6] Grupper M Kravtsov A Potasman I. Emphysematous cystitis: illustrative case report and review of the literature. Medicine. (2007) 86:47–53. 10.1097/MD.0b013e3180307c3a 17220755

[7] Kandefer-Gola M Ciaputa R Nowak M Pozniak A Jaskólska A Pozniak B. Emphysematous cystitis in a dog: a case report. Med Weter. (2012) 68:501–3.

[8] Lippi I Mannucci T Santa DD Barella G Oranges M Citi S. Emphysematous cystitis: retrospective evaluation of predisposing factors and ultrasound features in 36 dogs and 2 cats. Can Vet J. (2019) 60:514–8. 31080265 PMC6463776

[9] Michaeli J Mogle P Perlberg S Heiman S Caine M. Emphysematous pyelonephritis. J Urol. (1984) 131:203–8. 10.1016/S0022-5347(17)50309-2 6366247

[10] Schicho A Stroszczynski C Wiggermann P. Emphysematous cystitis: mortality, risk factors, and pathogens of a rare disease. Clin Pract. (2017) 7:930. 10.4081/cp.2017.930 28567236 PMC5432942

[11] Moon R Biller DS Smee NM. Emphysematous cystitis and pyelonephritis in a nondiabetic dog and a diabetic cat. J Am Anim Hosp Assoc. (2014) 50:124–9. 10.5326/JAAHA-MS-5972 24446401

[12] Sadek AR Blake H Mehta A. Emphysematous cystitis with clinical subcutaneous emphysema. Int J Emerg Med. (2011) 4:26. 10.1186/1865-1380-4-26 21668949 PMC3123544

[13] Spaulding CN Klein RD Ruer S Kau AL Schreiber HL Cusumano ZT. Selective depletion of uropathogenic *E. coli* from the gut by a FimH antagonist. Nature. (2017) 546:528–32. 10.1038/nature22972 28614296 PMC5654549

[14] Amano M Shimizu T. Emphysematous cystitis: a review of the literature. Intern Med. (2014) 53:79–82. 10.2169/internalmedicine.53.1121 24429444

[15] Huang JJ Chen KW Ruaan MK. Mixed acid fermentation of glucose as a mechanism of emphysematous urinary tract infection. J Urol. (1991) 146:148–51. 10.1016/S0022-5347(17)37736-4 2056576

[16] Fumeo M Manfredi S Volta A. Emphysematous cystitis: review of current literature, diagnosis and management challenges. Vet Med. (2019) 10:77–83. 10.2147/VMRR.S210463 31440461 PMC6679702

[17] Lapides J. Mechanisms of urinary tract infection. Urology. (1979) 14:217–25. 10.1016/0090-4295(79)90486-2 384642

[18] Thomas AA Lane BR Thomas AZ Remer EM Campbell SC Shoskes DA. Emphysematous cystitis: a review of 135 cases. BJU Int. (2007) 100:17–20. 10.1111/j.1464-410X.2007.06930.x 17506870

[19] Eken A Alma E. Emphysematous cystitis: the role of CT imaging and appropriate treatment. Can Urol Assoc J. (2013) 7:E754–6. 10.5489/cuaj.472 24282470 PMC3840514

[20] Fabbi M Manfredi S Bianchi E Gnudi G Miduri F Volta A. Emphysematous pyelitis and cystitis associated with vesicoureteral reflux in a diabetic dog. Can Vet J. (2016) 57:382–6. 27041755 PMC4790229

[21] Merkel LK Lulich J Polzin D Ober C Westropp J Sykes J. Clinicopathologic and microbiologic findings associated with emphysematous cystitis in 27 dogs. J Am Anim Hosp Assoc. (2017) 53:313–20. 10.5326/JAAHA-MS-6722 28892422

